# Slow-wave sleep as a key player in offline memory processing: insights from human EEG studies

**DOI:** 10.3389/fnbeh.2025.1620544

**Published:** 2025-08-06

**Authors:** Leanna Keeble, Padraic Monaghan, Edwin M. Robertson, Sana Hannan

**Affiliations:** ^1^Department of Biomedical and Life Sciences, Lancaster University, Lancaster, United Kingdom; ^2^Department of Psychology, Lancaster University, Lancaster, United Kingdom; ^3^Centre for Cognitive Neuroimaging, University of Glasgow, Glasgow, United Kingdom

**Keywords:** slow-wave sleep (SWS), EEG, sleep microstructure, memory consolidation, slow oscillations, sleep spindles, sleep disruption, therapeutic interventions

## Abstract

Slow-wave sleep (SWS) plays a pivotal role in memory consolidation, and electroencephalography (EEG) has provided critical insights into the neural mechanisms underlying these processes. In this mini-review, we discuss how SWS supports the processing of both declarative and procedural memory, in addition to higher cognitive functioning. We focus on the latest evidence from human EEG studies that examine temporal regularities alongside those that have demonstrated the coordinated interplay between slow oscillations, sleep spindles, and hippocampal ripples. We discuss how the precise temporal coupling of these oscillatory events facilitates memory transfer from the hippocampus to the neocortex, enhancing neuronal reactivation and optimizing long-term memory consolidation. We also examine how disruptions to SWS—due to lifestyle factors, ageing, neurological disorders, or pharmacological agents—can impair slow-wave activity and spindle dynamics, leading to memory deficits. Further, we highlight emerging neuromodulation techniques, such as transcranial direct current stimulation and closed-loop auditory stimulation, which harness EEG-based insights to enhance SWS and improve memory outcomes. These findings collectively demonstrate the potential of integrating EEG methodologies with targeted therapeutic interventions to restore SWS, optimize memory consolidation and enhance cognitive health. Finally, we recommend directions for future research aimed at refining these approaches, evaluating their long-term efficacy across diverse populations, and exploring new strategies to preserve memory function in the context of healthy ageing and neurological disease.

## Introduction

Sleep plays a critical role in offline memory processing and impacts how memories are consolidated. Memory consolidation refers to the stabilization of newly encoded information, its transfer from short-term to long-term storage, and its integration into existing memory networks (Alberini et al., 2013; Breton and Robertson, 2013). Slow-wave sleep (SWS) in particular has been linked with facilitating this consolidation (Gais and Born, 2004; Marshall and Born, 2007; Prehn-Kristensen et al., 2014; Sopp et al., 2018). This mini-review examines recent evidence on the role of SWS, with a focus on its microstructure (e.g., slow oscillations, spindles, and their coupling) over macrostructure (e.g., total time spent in SWS) on declarative and procedural memory, as well as higher order cognitive functions, examined through the lens of human electroencephalography (EEG). A deeper understanding of these precise mechanisms is crucial for the development of targeted interventions to optimize sleep and enhance memory consolidation.

SWS is characterized by high-amplitude slow waves that occur at a frequency of 0.5–4 Hz, including slow oscillations (0.5–1 Hz), which have a peak-to-peak amplitude of ≥75 μV (Spriggs, 2010; Mölle and Born, 2011a,b; Troester et al., 2023). Slow oscillations represent synchronized neuronal activity alternating between up-states (neuronal depolarization) and down-states (neuronal hyperpolarization) (Farhadian et al., 2021; Menicucci et al., 2020). Sleep spindles, brief (<500 ms) bursts of rhythmic activity at 11–16 Hz, are also observed in SWS and are closely linked to sigma power in the EEG (12–16 Hz), with higher sigma power indicating higher spindle rates (Spriggs, 2010; Troester et al., 2023). While spindles typically occur during N2 sleep, this mini-review will focus on those seen in SWS which may be more critical for memory consolidation (Staresina et al., 2023). This is discussed further in the ‘The role of slow-wave sleep in declarative memory’.

These oscillations are typically measured using EEG, a non-invasive technique that records the synchronous electrical activity of cortical neurons via electrodes placed on the scalp (Nayak and Anilkumar, 2025). EEG offers a high temporal resolution, enabling real-time observation and precise tracking of neural oscillations across a broad range of frequencies.

SWS has been primarily associated with both the consolidation of declarative memory, the system responsible for learning facts and events (Ackermann and Rasch, 2014; Marshall and Born, 2007; Plihal and Born, 1997), and higher cognitive processing (Lerner and Gluck, 2019). However, growing evidence suggests that SWS also contributes to procedural memory consolidation, including the acquisition of motor skills (Astill et al., 2014; Cousins et al., 2014). Sleep disruptions can interfere with SWS microstructure, thereby impairing memory consolidation and subsequent cognitive functioning. Such disruptions are becoming increasingly prevalent due to modern lifestyles, driven by factors such as exposure to blue light-emitting devices (Ishizawa et al., 2021), the use of pharmaceuticals for neurological disorders such as anti-seizure medications for epilepsy (Roebber et al., 2022), mental health conditions including depression (Riemann et al., 2020), and age-related changes (Hornung et al., 2005). Therefore, understanding how these diverse disruptions differentially impact SWS-related memory processes is essential for informing strategies that preserve cognitive health across the lifespan, in both clinical and healthy populations, and mitigate the growing societal burden of memory-related impairments.

Human EEG studies have considerably advanced our understanding of the neurophysiological mechanisms underlying sleep-dependent memory consolidation. This mini-review will highlight the central role of slow oscillations and their coordination with sleep spindles and hippocampal ripples in facilitating hippocampo-neocortical communication – processes critical for memory consolidation. We will also discuss higher cognitive processes and how disruptions to SWS can adversely impact memory performance, underscoring the importance of this sleep stage in offline memory processing. Finally, we will examine emerging therapeutic interventions designed to mitigate SWS disruptions and enhance memory consolidation, highlighting the potential of targeting SWS to improve memory and cognitive function.

### The role of slow-wave sleep in declarative memory

Declarative memory refers to the ability to recall facts and events and can be categorized into two subtypes: semantic memory (facts) and episodic memory (events) (Tulving, 1972). SWS has long been recognized as playing a key role in the consolidation and retrieval of declarative memories (Höller et al., 2024; Plihal and Born, 1997; Prehn-Kristensen et al., 2014; Zohuri and McDaniel, 2022). Retrieval refers to the process of accessing stored information from long-term memory (Gilboa, 2015). During wakefulness, representations of newly acquired declarative memories are initially encoded within the hippocampus (Gais and Born, 2004; Marshall et al., 2004; Sopp et al., 2018). These memories are then replayed and reactivated through coordinated hippocampal neural firing during SWS, facilitating their transfer from a fragile, hippocampus-dependent state to more stable neocortical storage (Cox et al., 2020; Fogel et al., 2007; Siapas and Wilson, 1998). EEG studies have demonstrated that neuronal reactivation during SWS following declarative learning strengthens memory consolidation by supporting this hippocampo-neocortical transfer (Gais and Born, 2004; Mölle & Born, 2011a,b). Critically, this process is mediated by the precise temporal coupling between slow oscillations and sleep spindles, which orchestrate neuronal reactivation and synaptic plasticity, thereby enhancing consolidation (Hahn et al., 2022; Hong et al., 2025; Joechner et al., 2023; Mölle & Born, 2011a,b; Sánchez-Corzo et al., 2024). This synchrony reflects the reprocessing of newly acquired information, with coherence between slow oscillations and spindles increasing during periods of sleep undertaken within an hour of intensive declarative learning (Zohuri and McDaniel, 2022). A potential explanation for this evidence of temporal coupling is that SWS provides an optimal neurochemical environment for memory reprocessing (Gais and Born, 2004; Zohuri and McDaniel, 2022). Elevated cholinergic tone has been suggested to support memory encoding, but if not downregulated during sleep, it may interfere with the consolidation of hippocampus-dependent declarative memories (Gais and Born, 2004). Similarly, elevated cortisol levels exert an inhibitory effect on hippocampal feedback mechanisms, further disrupting memory consolidation (McAuley et al., 2009).

Spindles that arise in SWS have been proposed to play a more important role in memory consolidation than those found in N2 sleep due to temporal coupling (Staresina et al., 2023). This highlights an important distinction around the functional significance of isolated spindles in N2 versus SWS-coupled spindles, as it suggests that the timing and coordination of spindles with other oscillations, rather than their absolute quantity, may be more critical for memory. A key function of N2 spindles is their role in raising arousal thresholds and shielding the sleeping brain from external stimuli, thereby serving as a protective factor from awakenings (Dang-Vu et al., 2010; Fernandez and Lüthi, 2019). They have also been consistently associated with local cortical plasticity and are considered a physiological index of trait-like cognitive abilities and experience-dependent learning rather than in systems-level memory transfer (Barakat et al., 2013; Fernandez and Lüthi, 2019; Fogel and Smith, 2011). While memory consolidation may involve bidirectional interactions between sleep microstructure and neural reactivation, evidence from transcranial and auditory stimulation supports the view that the coordination between slow oscillations and spindles during SWS plays an active, mechanistic role in facilitating memory consolidation (Marshall et al., 2006; Marshall et al., 2004; Ngo et al., 2013).

Slow oscillations help coordinate thalamocortical spindle activity by providing depolarizing up-states, which are reflected in surface-positive field potentials. These up-states promote cortical excitability and create favorable conditions for the synaptic plasticity necessary for long-term memory formation (Gais and Born, 2004; Mölle et al., 2002). This is supported by recent studies which employed intracranial EEG where depth electrodes were stereotactically implanted into the brain of epilepsy patients to localize seizure foci (Dasgupta et al., 2022; Staresina et al., 2023). These studies demonstrate that thalamic spindles, facilitated by slow oscillation up-states, organize the occurrence of ripples which establish the optimal conditions required for synaptic plasticity and consolidation (Staresina et al., 2023). EEG studies using transcranial direct current stimulation (tDCS) during SWS have further emphasized its critical role in declarative memory formation. A study utilizing anodal tDCS of frontocortical regions during SWS-rich sleep demonstrated that stimulation was associated with greater slow-wave activity (<3 Hz), and increased retention of word pairs (Marshall et al., 2004). This is further supported by evidence displaying that the interplay between slow oscillations and spindles during SWS – specifically, the coordination between maximum spindle amplitude and slow oscillation up-states – was marked by significant improvement in declarative memory consolidation (Mikutta et al., 2019). In summary, the mechanisms underlying SWS – including reactivation processes, temporal coordination between slow oscillations and spindles, and optimal neurochemical environments – play a crucial role in facilitating the transfer and integration of declarative memories into long-term storage.

### The role of slow-wave sleep in procedural memory

While procedural memory has been more closely associated with rapid eye movement (REM) sleep (Marshall and Born, 2007), emerging research suggests that SWS also plays a critical role in its consolidation (Fogel et al., 2007; Höller et al., 2024). Both slow-wave activity and spindle-related sigma power contribute to enhancing motor skill learning (Fogel et al., 2007; Holz et al., 2012). Sigma power is associated with memory reactivation and processes supporting synaptic plasticity during sleep (Menicucci et al., 2020; Xia et al., 2023). This is particularly important as sleep spindles are crucial for facilitating communication between the motor cortex and subcortical structures such as the basal ganglia (Barakat et al., 2013; Holz et al., 2012; Salih et al., 2009). Moreover, higher sigma power has been linked to enhanced performance in procedural memory tasks (Holz et al., 2012). Spindle-related sigma activity may therefore promote neuronal reactivation during sleep, thereby enhancing procedural memory consolidation. Further evidence has demonstrated that enhanced slow-wave and sigma activity after motor learning is associated with superior skill retention (Holz et al., 2012; Poon et al., 2019).

**Figure 1:**
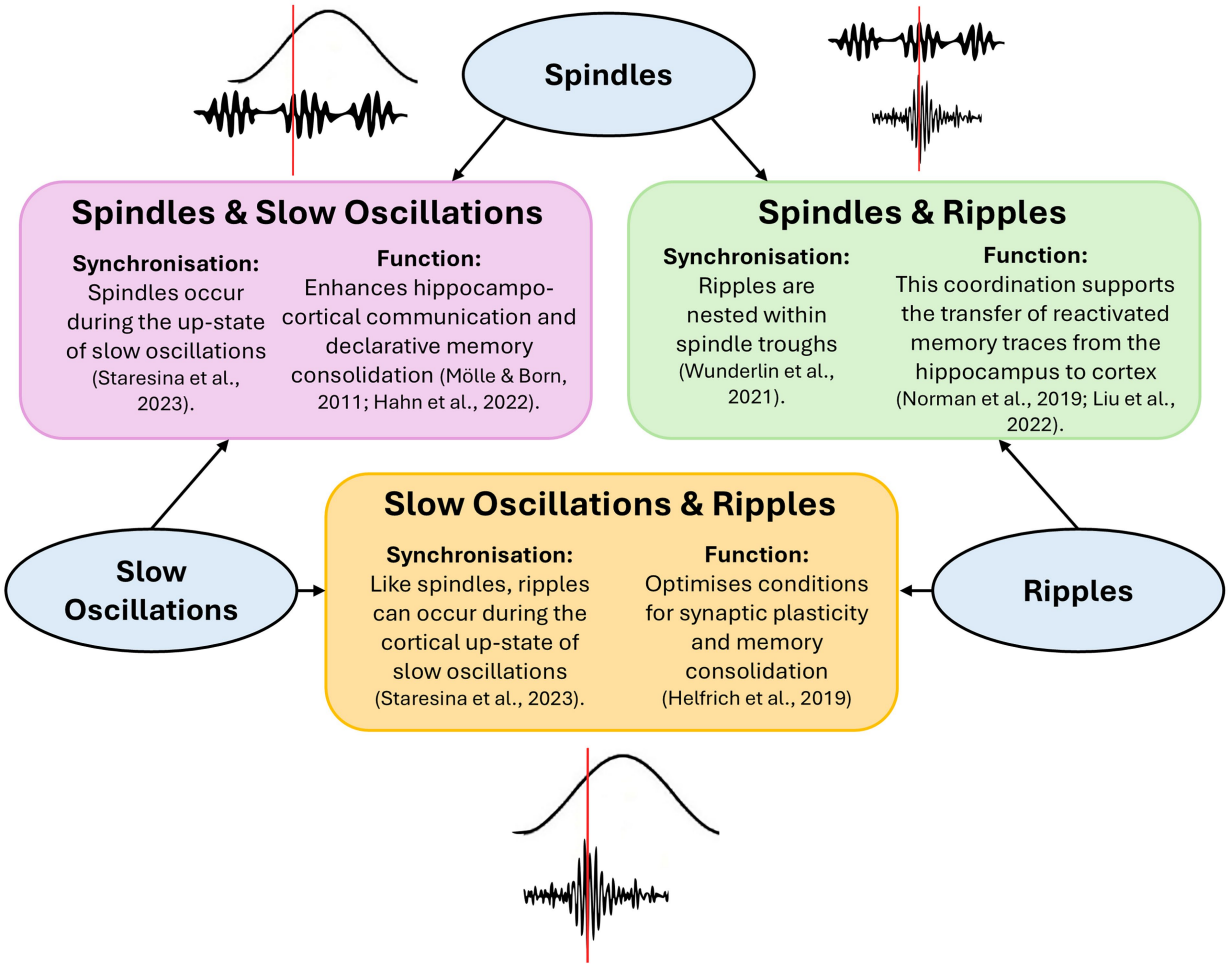
Interactions between slow oscillations, spindles, and ripples supporting memory consolidation during sleep. The diagram illustrates the temporal coordination between three key neural oscillations—slow oscillations, spindles, and ripples—and their roles in memory consolidation. Spindles occur during the up-state of slow oscillations, enhancing hippocampo-cortical communication. Ripples, nested within spindle troughs, facilitate the transfer of reactivated memory traces from hippocampus to cortex. Additionally, ripples can co-occur with the up-state of slow oscillations, optimizing synaptic plasticity and memory consolidation.

Sharp-wave ripples (SWRs) are also implicated in memory consolidation, particularly in the transfer of information from the hippocampus to the neocortex (Zhou and Norimoto, 2023). SWRs consist of two components: sharp waves, which are large, negative deflections in the local field potential generated by synchronized depolarization of hippocampal pyramidal neurons, and ripples, brief high-frequency oscillations (typically 110–180 Hz) superimposed on the sharp wave, thought to reflect coordinated neuronal firing (Hall and Wang, 2023; Liu et al., 2022; Norman et al., 2019). Ripples are often triggered by sharp waves and may support the replay of memory traces during sleep, facilitating their consolidation into long-term storage (Hall and Wang, 2023; Liu et al., 2022; Norman et al., 2019). SWRs in the hippocampus are nested within spindle troughs, and spindles are thought to coordinate the timing of these ripples to facilitate information transfer from the hippocampus to the neocortex (Wunderlin et al., 2021). This precise temporal coordination may support hippocampo-neocortical communication by aligning hippocampal output with periods of increased cortical excitability, thereby enhancing consolidation (Helfrich et al., 2019; Staresina et al., 2023; Wunderlin et al., 2021). Supporting this, fast spindles are more likely to occur following a period of motor learning, particularly during slow oscillation up-states, and slow oscillation-spindle coupling is in turn associated with improved memory consolidation (Solano et al., 2022). Mikutta et al. (2019) provided further evidence to support this by demonstrating that the strength of slow oscillation-spindle coupling, as measured by the modulation index, is correlated with procedural memory consolidation (Mikutta et al., 2019). In summary, there is a growing body of evidence supporting the coupling of spindles and slow oscillations in facilitating the consolidation of procedural memory, by coordinating cross-regional communication and optimizing temporal dynamics for memory processing (Figure 1).

### The role of slow-wave sleep in higher cognitive processing

In addition to its role in declarative and procedural memory consolidation, growing evidence suggests SWS also supports higher cognitive processes such as pattern extraction, rule learning, and insight. These functions rely on the integration of information across events, often involving temporal regularities and hippocampal–neocortical communication (Lerner and Gluck, 2019, 2022).

It has been suggested that hippocampal memories are replayed in a temporally compressed format during SWS, allowing for temporal patterns and connections to be identified during sleep that were separate during encoding (Lerner and Gluck, 2019). This is evidenced, for example, in the number reduction task, where participants are significantly more likely to gain explicit insight into a hidden temporal rule following SWS-rich sleep compared to equivalent periods of wakefulness (Wagner et al., 2004; Yordanova et al., 2008; Yordanova et al., 2012). Similar findings have also been reported across a range of other cognitive tasks. For instance, in the serial reaction time task, which is often used to examine procedural learning (Robertson et al., 2004), SWS has been shown to promote the transition from implicit to explicit knowledge of embedded sequences (Wilhelm et al., 2013). This is further supported by targeted memory reactivation during SWS, with performance improvements linked to SWS duration and slow oscillation-spindle coupling (Cousins et al., 2014; Diekelmann et al., 2016; Yordanova et al., 2017). Robertson et al. also highlighted the role of awareness in moderating the effect of sleep on sequence learning, suggesting that sleep may support explicit abstraction under specific conditions (Robertson et al., 2004).

Beyond motor learning, evidence from statistical learning paradigms further supports a role for SWS in abstraction. SWS has been shown to facilitate the extraction of probabilistic regularities across auditory streams (Durrant et al., 2013, 2016; Durrant et al., 2011). Additionally, reactivation during SWS has been shown to enhance the recognition of structured patterns, implicating offline reprocessing in the extraction of higher-order structure (Hennies et al., 2017). Similarly, studies using artificial grammar learning paradigms have demonstrated improved rule generalization following SWS, again suggesting that sleep supports the abstraction of latent structure from newly learned material (Gaskell et al., 2014).

Collectively, these findings indicate that SWS supports not only the stabilization of individual memory traces, but also the abstraction of temporal regularities and hidden rules across experiences that were not evident during wakeful encoding (Lewis and Durrant, 2011). Increased coordination between slow oscillations and sleep spindles during SWS may thus provide a critical mechanism for reorganizing hippocampal-dependent memories and promoting the emergence of higher-level cognitive processing.

### Disruptions to slow-wave sleep and their impact on memory

**Figure 2:**
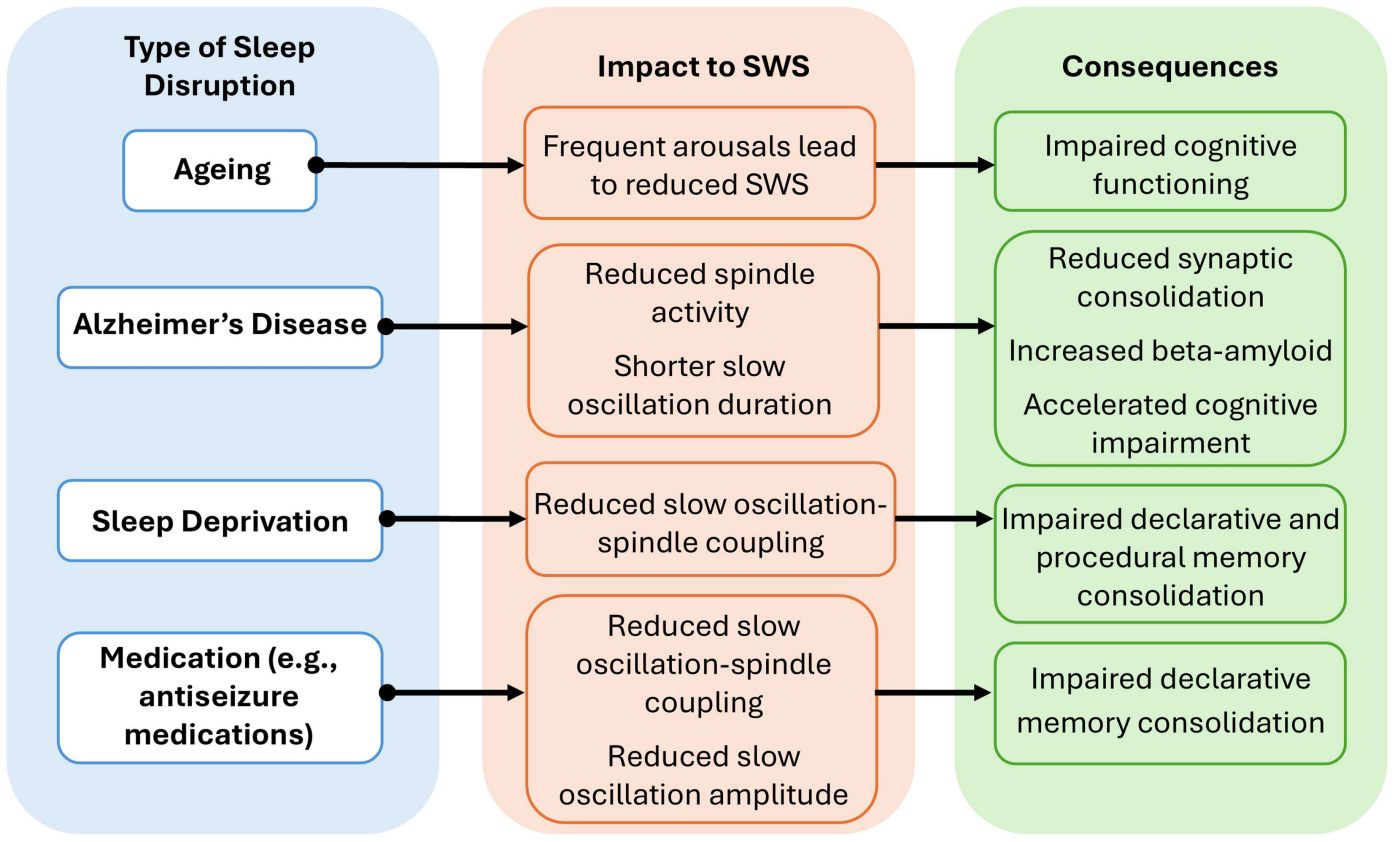
Overview of common sleep disruptions and their impact on slow-wave sleep (SWS) and memory. Four major sources of sleep disruption are highlighted, including how they impact SWS microstructure and the subsequent effects on memory and cognitive health.

Sleep duration and quality can be disrupted by various factors, including modern lifestyle pressures (Ishizawa et al., 2021), ageing (Hornung et al., 2005), neurological conditions like Alzheimer’s disease (AD) (Hanert et al., 2024) and epilepsy (van Schalkwijk et al., 2018), and pharmaceutical interventions (Roebber et al., 2022) (Figure 2). Poor sleep quality is associated with reduced hippocampal volume (Fjell et al., 2020), which may in turn impair declarative memory performance, given that greater hippocampal volume has been linked to better outcomes on tasks such as the California Verbal Learning Test (Pohlack et al., 2014). Sleep deprivation and disruption can be side effects of modern work-life pressures, with a growing proportion of the population failing to meet the recommended minimum of 7 h sleep per night (Watson et al., 2015). Recent reports indicate that approximately 30% of adults regularly obtain less than this recommended amount, and only 15% achieve this minimum across five consecutive nights (Scott et al., 2024). This is substantial as sleep deprivation significantly attenuates slow oscillation-spindle coupling, leading to impaired declarative and procedural memory retention (Snipes et al., 2022). Sleep loss has been shown to impair SWS and disrupt the balance of cortical excitability necessary for memory integration (Abel et al., 2013). This disruption is linked to increased theta activity (4–8 Hz), which reflects a slowing of neural processing and reduced cortical responsiveness (Magnuson et al., 2022; Snipes et al., 2022). Elevated theta power can persist for up to 24 h after sleep deprivation and is associated with poorer performance on visuomotor memory tasks (Magnuson et al., 2022). Additionally, reduced cortical inhibition during heightened theta activity may further interfere with memory-related neural dynamics, suggesting that sleep loss can alter the functional readiness of the brain for memory processing (Snipes et al., 2022).

Given our ageing population, it is becoming increasingly important to consider age-related changes to SWS and memory (World Health Organisation, 2024). Ageing is associated with increased rates of overnight arousals, reducing SWS quantity and impairing cognitive functioning (Spriggs, 2010; Zeller et al., 2024). This is particularly concerning given that a 1% reduction in SWS is linked to a 27% increased risk of dementia (Himali et al., 2023). Individuals with neurodegenerative diseases such as AD often exhibit pronounced SWS deficits, which contribute to a bidirectional cycle where cognitive decline can worsen sleep disturbances, and disrupted sleep further exacerbates cognitive impairment (Himali et al., 2023; Zeller et al., 2024). EEG studies demonstrate that patients with AD have significantly reduced spindle amplitude and shorter slow oscillation duration compared to healthy adults, which is believed to disrupt hippocampus-dependent memory consolidation (Hanert et al., 2024). Additionally, disruptions to SWS may underlie reduced synaptic consolidation in these individuals (Joechner et al., 2023). SWS quantity begins to decline around the age of 60 and is linked to increased risk of AD amongst other dementias (Himali et al., 2023). This association is thought to reflect the role of SWS in clearing metabolic waste from the brain, including the protein amyloid-β, a hallmark of AD (Himali et al., 2023). These findings illustrate the varied and critical role of SWS in maintaining cognitive health across the lifespan.

The importance of SWS is further highlighted when considering other neurological conditions, such as epilepsy (van Schalkwijk et al., 2018). Nocturnal seizures lead to a significant decline in memory retention rates, dropping from 92% to 60% (Sarkis et al., 2016). In addition, interictal epileptiform discharges originating in the hippocampus during sleep have been shown to impact long-term declarative memory consolidation (Lambert et al., 2021). Anti-seizure medications themselves can also have a detrimental impact on the slow oscillation-spindle coupling necessary for memory consolidation and integration (Motamedi and Meador, 2004; Roebber et al., 2022). When examining SWS microstructure in a heterogeneous cohort of individuals with epilepsy after administering different anti-seizure medications, EEG recordings showed a reduction in both slow oscillation-spindle coupling and slow oscillation amplitude (Roebber et al., 2022). Moreover, a reduction in medication showed improvements to declarative memory (Durwen and Elger, 1993; Motamedi and Meador, 2004).

### Targeting slow-wave sleep: therapeutic interventions to improve memory

Considering the critical role SWS plays in memory, several therapeutic strategies have been developed to enhance its function. Neuromodulation techniques such as tDCS and closed-loop auditory stimulation have been shown to increase slow-wave activity and improve memory performance (Marshall et al., 2004; Wunderlin et al., 2021), while pharmacological and behavioral strategies can also be used to manipulate SWS (Cross et al., 2025; Rasch and Born, 2013). Marshall et al. (2004) applied anodal tDCS during the first sleep cycle, a period rich in SWS but relatively low in REM. While declarative memory was significantly enhanced under these conditions, procedural memory showed no improvement. The authors proposed that this may be due to the use of a mirror-tracing task, which could rely more heavily on REM sleep. However, this null result contrasts with literature suggesting that procedural memory can benefit from SWS (Fogel et al., 2007; Höller et al., 2024). Transcranial oscillating direct current stimulation (toDCS) targeting SWS has shown similar effects to tDCS. In children with ADHD, whose declarative memory consolidation is typically impaired, toDCS can restore memory performance to levels comparable to healthy controls (Prehn-Kristensen et al., 2014). These contradictory findings highlight the need for further research to characterize the stage- and task-specificity of procedural memory consolidation, as well as to refine the stimulation parameters of neuromodulatory techniques — including spatial targeting, frequency, amplitude, and timing — that may enhance their effects.

Phase-locked acoustic stimulation is another non-invasive technique whereby short acoustic stimuli are delivered, synchronized to slow wave peaks (Zeller et al., 2024). This increases slow-wave activity, as well as spindle power and delta and theta oscillations, resulting in improved memory performance, even in older adults with cognitive impairments (Zeller et al., 2024). While still under development, this technique shows potential for application in individuals with cognitive impairments, such as those with AD (Wunderlin et al., 2021). Another novel therapeutic approach is closed-loop vibration stimulation, which delivers brief vibratory pulses synchronized with the individual’s heart rate (Choi et al., 2021). This was shown to enhance the depth of SWS, as seen in EEG spectral power and frequency measures, and to improve declarative and motor learning through slow-wave activity, slow oscillation-spindle coupling, and theta power (Choi et al., 2021; Hahn et al., 2022).

Pharmaceutical interventions have been used to target neuromodulatory systems such as the cholinergic system to manipulate SWS microstates and promote memory consolidation. Cholinergic activation is highest during wakefulness (Marshall and Born, 2007) and naturally low after periods of declarative learning (Zohuri and McDaniel, 2022). Experimental studies using cholinergic antagonists have demonstrated that blocking cholinergic activity promotes declarative memory consolidation, while procedural memory is largely unaffected (Gais and Born, 2004; Rasch and Born, 2013).

Finally, behavioral strategies such as targeted memory reactivation (TMR) have been proposed to selectively reinforce memory traces during SWS (Cross et al., 2025). This involves pairing learning with auditory or olfactory stimuli, with re-exposure to cues during SWS shown to increase hippocampal activation (Marshall and Born, 2007), spindle rates and duration, and slow wave amplitude (Sánchez-Corzo et al., 2024), to facilitate memory consolidation (Marshall and Born, 2007). Given the promising results of these therapeutic interventions, future research should aim to refine or combine these approaches to optimize memory consolidation — not only in individuals with cognitive impairments, but also across the wider population.

## Conclusion

In summary, we have highlighted the critical and multifaceted role of SWS in offline memory processing, particularly in the consolidation of both declarative and procedural memory and higher order cognitive functions. Declarative memory consolidation during SWS is driven by reactivation of hippocampal neural firing, a process facilitated by the synchronized interplay of slow oscillations and sleep spindles, as well as the suppression of cholinergic tone and cortisol feedback. In contrast, procedural memory consolidation is closely linked to sigma activity and precise temporal coupling between spindles and slow oscillations, which optimize conditions for synaptic plasticity and support the coordination of cross-regional communication. Additionally, SWS has been demonstrated to extend its role beyond memory consolidation itself to support the abstraction of patterns, rules, and structure across experiences. Through hippocampal-neocortical interactions and temporally compressed replay, SWS facilitates the integration and transformation of memories, enabling the emergence of higher-order cognitive insight.

Advancing our understanding of how SWS supports memory consolidation and how this can be effectively enhanced holds significant potential for developing therapies that enhance cognitive resilience in ageing and neurodegenerative diseases. As such, a key recommendation for future research is to combine normative EEG-based mapping of SWS with behavioral assessments across memory systems to distinguish healthy ageing from pathological sleep disruptions. This approach could further guide the development of targeted interventions to preserve and improve cognitive function across the lifespan and promote healthy ageing.

## References

[ref1] Abel T. Havekes R. Saletin J. M. Walker M. P. (2013). Sleep, plasticity and memory from molecules to whole-brain networks. Curr. Biol. 23, R774–R788. doi: 10.1016/j.cub.2013.07.025, PMID: 24028961 PMC4263505

[ref2] Ackermann S. Rasch B. (2014). Differential effects of non-REM and REM sleep on memory consolidation? Curr. Neurol. Neurosci. Rep. 14:430. doi: 10.1007/s11910-013-0430-8, PMID: 24395522

[ref3] Alberini C. M. Johnson S. A. Ye X. (2013). “Chapter five - memory reconsolidation: lingering consolidation and the dynamic memory trace” in Memory reconsolidation. ed. Alberini C. M. (Academic Press), 81–117.

[ref4] Astill R. G. Piantoni G. Raymann R. J. Vis J. C. Coppens J. E. Walker M. P.. (2014). Sleep spindle and slow wave frequency reflect motor skill performance in primary school-age children. Front. Hum. Neurosci. 8:910. doi: 10.3389/fnhum.2014.00910, PMID: 25426055 PMC4227520

[ref5] Barakat Carrier Debas Lungu Fogel Vandewalle. (2013). Sleep spindles predict neural and behavioral changes in motor sequence consolidation. Hum. Brain Mapp. 34, 2918–2928. doi: 10.1002/hbm.22116 22674673 PMC6870513

[ref6] Breton J. Robertson E. M. (2013). Memory processing: the critical role of neuronal replay during sleep. Curr. Biol. 23, R836–R838. doi: 10.1016/j.cub.2013.07.068, PMID: 24070442

[ref7] Choi S. H. Kwon H. B. Jin H. W. Yoon H. Lee M. H. Lee Y. J.. (2021). Weak closed-loop vibrational stimulation improves the depth of slow-wave sleep and declarative memory consolidation. Sleep 44. doi: 10.1093/sleep/zsaa285, PMID: 33367712

[ref8] Cousins J. N. El-Deredy W. Parkes L. M. Hennies N. Lewis P. A. (2014). Cued memory reactivation during slow-wave sleep promotes explicit knowledge of a motor sequence. J. Neurosci. 34, 15870–15876. doi: 10.1523/jneurosci.1011-14.2014, PMID: 25429129 PMC4244461

[ref9] Cox R. Rüber T. Staresina B. P. Fell J. (2020). Phase-based coordination of hippocampal and neocortical oscillations during human sleep. Commun Biol 3:176. doi: 10.1038/s42003-020-0913-5, PMID: 32313064 PMC7170909

[ref10] Cross Z. R. Helfrich R. F. Corcoran A. W. Dede A. J. O. Kohler M. J. Coussens S. W.. (2025). Slow oscillation-spindle coupling predicts sequence-based language learning. J. Neurosci. 45:e2193232024. doi: 10.1523/jneurosci.2193-23.2024, PMID: 39572236 PMC11735671

[ref11] Dang-Vu T. T. Schabus M. Desseilles M. Sterpenich V. Bonjean M. Maquet P. (2010). Functional neuroimaging insights into the physiology of human sleep. Sleep 33, 1589–1603. doi: 10.1093/sleep/33.12.1589, PMID: 21120121 PMC2982729

[ref12] Dasgupta D. Miserocchi A. McEvoy A. W. Duncan J. S. (2022). Previous, current, and future stereotactic EEG techniques for localising epileptic foci. Expert Rev. Med. Devices 19, 571–580. doi: 10.1080/17434440.2022.2114830, PMID: 36003028 PMC9612928

[ref13] Diekelmann S. Born J. Rasch B. (2016). Increasing explicit sequence knowledge by odor cueing during sleep in men but not women. Front. Behav. Neurosci. 10:74. doi: 10.3389/fnbeh.2016.00074 27147995 PMC4828435

[ref14] Durrant S. J. Cairney S. A. Lewis P. A. (2013). Overnight consolidation aids the transfer of statistical knowledge from the medial temporal lobe to the striatum. Cereb. Cortex 23, 2467–2478. doi: 10.1093/cercor/bhs244, PMID: 22879350

[ref15] Durrant S. J. Cairney S. A. Lewis P. A. (2016). Cross-modal transfer of statistical information benefits from sleep. Cortex 78, 85–99. doi: 10.1016/j.cortex.2016.02.011, PMID: 27017231

[ref16] Durrant S. J. Taylor C. Cairney S. Lewis P. A. (2011). Sleep-dependent consolidation of statistical learning. Neuropsychologia 49, 1322–1331. doi: 10.1016/j.neuropsychologia.2011.02.015, PMID: 21335017

[ref17] Durwen H. F. Elger C. E. (1993). Verbal learning differences in epileptic patients with left and right temporal lobe foci--a pharmacologically induced phenomenon? Acta Neurol. Scand. 87, 1–8. doi: 10.1111/j.1600-0404.1993.tb04066.x, PMID: 8424306

[ref18] Farhadian N. Khazaie H. Nami M. Khazaie S. (2021). The role of daytime napping in declarative memory performance: a systematic review. Sleep Med. 84, 134–141. doi: 10.1016/j.sleep.2021.05.019, PMID: 34148000

[ref19] Fernandez L. M. J. Lüthi A. (2019). Sleep spindles: mechanisms and functions. Physiol. Rev. 100, 805–868. doi: 10.1152/physrev.00042.2018 31804897

[ref20] Fjell A. M. Sørensen Ø. Amlien I. K. Bartrés-Faz D. Bros D. M. Buchmann N.. (2020). Self-reported sleep relates to hippocampal atrophy across the adult lifespan: results from the Lifebrain consortium. Sleep 43:280. doi: 10.1093/sleep/zsz280, PMID: 31738420 PMC7215271

[ref21] Fogel S. M. Smith C. T. (2011). The function of the sleep spindle: a physiological index of intelligence and a mechanism for sleep-dependent memory consolidation. Neurosci. Biobehav. Rev. 35, 1154–1165. doi: 10.1016/j.neubiorev.2010.12.003, PMID: 21167865

[ref22] Fogel S. M. Smith C. T. Cote K. A. (2007). Dissociable learning-dependent changes in REM and non-REM sleep in declarative and procedural memory systems. Behav. Brain Res. 180, 48–61. doi: 10.1016/j.bbr.2007.02.037, PMID: 17400305

[ref23] Gais S. Born J. (2004). Declarative memory consolidation: mechanisms acting during human sleep. Learn. Mem. 11, 679–685. doi: 10.1101/lm.80504, PMID: 15576885 PMC534696

[ref24] Gaskell M. G. Warker J. Lindsay S. Frost R. Guest J. Snowdon R.. (2014). Sleep underpins the plasticity of language production. Psychol. Sci. 25, 1457–1465. doi: 10.1177/0956797614535937, PMID: 24894583

[ref25] Gilboa A. (2015). “Retrieval” in International Encyclopedia of the Social & Behavioral Sciences. ed. Wright J. D.. Second ed (Orlando, FL: Elsevier), 608–612.

[ref26] Hahn M. A. Bothe K. Heib D. Schabus M. Helfrich R. F. Hoedlmoser K. (2022). Slow oscillation-spindle coupling strength predicts real-life gross-motor learning in adolescents and adults. eLife 11:66761. doi: 10.7554/eLife.66761, PMID: 35188457 PMC8860438

[ref27] Hall A. F. Wang D. V. (2023). The two tales of hippocampal sharp-wave ripple content: the rigid and the plastic. Prog. Neurobiol. 221:102396. doi: 10.1016/j.pneurobio.2022.102396, PMID: 36563928 PMC9899323

[ref28] Hanert A. Schönfeld R. Weber F. D. Nowak A. Döhring J. Philippen S.. (2024). Reduced overnight memory consolidation and associated alterations in sleep spindles and slow oscillations in early Alzheimer's disease. Neurobiol. Dis. 190:106378. doi: 10.1016/j.nbd.2023.106378, PMID: 38103701

[ref29] Helfrich R. F. Lendner J. D. Mander B. A. Guillen H. Paff M. Mnatsakanyan L.. (2019). Bidirectional prefrontal-hippocampal dynamics organize information transfer during sleep in humans. Nat. Commun. 10:3572. doi: 10.1038/s41467-019-11444-x, PMID: 31395890 PMC6687745

[ref30] Hennies N. Lambon Ralph M. A. Durrant S. J. Cousins J. N. Lewis P. A. (2017). Cued memory reactivation during SWS abolishes the beneficial effect of sleep on abstraction. Sleep 40:zsx102. doi: 10.1093/sleep/zsx102, PMID: 28821209

[ref31] Himali J. J. Baril A. A. Cavuoto M. G. Yiallourou S. Wiedner C. D. Himali D.. (2023). Association between slow-wave sleep loss and incident dementia. JAMA Neurol. 80, 1326–1333. doi: 10.1001/jamaneurol.2023.3889, PMID: 37902739 PMC10616771

[ref32] Höller Y. Eyjólfsdóttir S. Van Schalkwijk F. J. Trinka E. (2024). The effects of slow wave sleep characteristics on semantic, episodic, and procedural memory in people with epilepsy. Front. Pharmacol. 15:1374760. doi: 10.3389/fphar.2024.1374760, PMID: 38725659 PMC11079234

[ref33] Holz J. Piosczyk H. Feige B. Spiegelhalder K. Baglioni C. Riemann D.. (2012). EEG Σ and slow-wave activity during NREM sleep correlate with overnight declarative and procedural memory consolidation. J. Sleep Res. 21, 612–619. doi: 10.1111/j.1365-2869.2012.01017.x, PMID: 22591117

[ref34] Hong X. Farmer C. Kozhemiako N. Holmes G. L. Thompson L. Manwaring S.. (2025). Differences in sleep EEG coherence and spindle metrics in toddlers with and without receptive/expressive language delay: a prospective observational study. J. Neurodev. Disord. 17:11. doi: 10.1186/s11689-024-09586-1, PMID: 39987026 PMC11847392

[ref35] Hornung O. P. Danker-Hopfe H. Heuser I. (2005). Age-related changes in sleep and memory: commonalities and interrelationships. Exp. Gerontol. 40, 279–285. doi: 10.1016/j.exger.2005.02.001, PMID: 15820608

[ref36] Ishizawa M. Uchiumi T. Takahata M. Yamaki M. Sato T. (2021). Effects of pre-bedtime blue-light exposure on ratio of deep sleep in healthy young men. Sleep Med. 84, 303–307. doi: 10.1016/j.sleep.2021.05.046, PMID: 34217920

[ref37] Joechner A. K. Hahn M. A. Gruber G. Hoedlmoser K. Werkle-Bergner M. (2023). Sleep spindle maturity promotes slow oscillation-spindle coupling across child and adolescent development. eLife 12:83565. doi: 10.7554/eLife.83565, PMID: 37999945 PMC10672804

[ref38] Lambert I. Tramoni-Negre E. Lagarde S. Pizzo F. Trebuchon-Da Fonseca A. Bartolomei F.. (2021). Accelerated long-term forgetting in focal epilepsy: do interictal spikes during sleep matter? Epilepsia 62, 563–569. doi: 10.1111/epi.16823, PMID: 33476422

[ref39] Lerner I. Gluck M. A. (2019). Sleep and the extraction of hidden regularities: a systematic review and the importance of temporal rules. Sleep Med. Rev. 47, 39–50. doi: 10.1016/j.smrv.2019.05.004, PMID: 31252335 PMC6779511

[ref40] Lerner I. Gluck M. A. (2022). Sleep facilitates extraction of temporal regularities with varying timescales [brief research report]. Front. Behav. Neurosci. 16:847083. doi: 10.3389/fnbeh.2022.847083 35401133 PMC8990849

[ref41] Lewis P. A. Durrant S. J. (2011). Overlapping memory replay during sleep builds cognitive schemata. Trends Cogn. Sci. 15, 343–351. doi: 10.1016/j.tics.2011.06.004, PMID: 21764357

[ref42] Liu A. A. Henin S. Abbaspoor S. Bragin A. Buffalo E. A. Farrell J. S.. (2022). A consensus statement on detection of hippocampal sharp wave ripples and differentiation from other fast oscillations. Nat. Commun. 13:6000. doi: 10.1038/s41467-022-33536-x, PMID: 36224194 PMC9556539

[ref43] Magnuson J. R. Kang H. J. Dalton B. H. McNeil C. J. (2022). Neural effects of sleep deprivation on inhibitory control and emotion processing. Behav. Brain Res. 426:113845. doi: 10.1016/j.bbr.2022.113845, PMID: 35304184

[ref44] Marshall L. Born J. (2007). The contribution of sleep to hippocampus-dependent memory consolidation. Trends Cogn. Sci. 11, 442–450. doi: 10.1016/j.tics.2007.09.001, PMID: 17905642

[ref45] Marshall L. Helgadóttir H. Mölle M. Born J. (2006). Boosting slow oscillations during sleep potentiates memory. Nature 444, 610–613. doi: 10.1038/nature05278, PMID: 17086200

[ref46] Marshall L. Mölle M. Hallschmid M. Born J. (2004). Transcranial direct current stimulation during sleep improves declarative memory. J. Neurosci. 24, 9985–9992. doi: 10.1523/jneurosci.2725-04.2004, PMID: 15525784 PMC6730231

[ref47] McAuley M. T. Kenny R. A. Kirkwood T. B. Wilkinson D. J. Jones J. J. Miller V. M. (2009). A mathematical model of aging-related and cortisol induced hippocampal dysfunction. BMC Neurosci. 10:26. doi: 10.1186/1471-2202-10-26, PMID: 19320982 PMC2680862

[ref48] Menicucci D. Piarulli A. Laurino M. Zaccaro A. Agrimi J. Gemignani A. (2020). Sleep slow oscillations favour local cortical plasticity underlying the consolidation of reinforced procedural learning in human sleep. J. Sleep Res. 29:e13117. doi: 10.1111/jsr.13117, PMID: 32592318

[ref49] Mikutta C. Feige B. Maier J. G. Hertenstein E. Holz J. Riemann D.. (2019). Phase-amplitude coupling of sleep slow oscillatory and spindle activity correlates with overnight memory consolidation. J. Sleep Res. 28:e12835. doi: 10.1111/jsr.12835, PMID: 30848042

[ref50] Mölle M. Born J. (2011a). “Chapter 7 - slow oscillations orchestrating fast oscillations and memory consolidation” in Progress in brain research. eds. Van Someren E. J. W. Van Der Werf Y. D. Roelfsema P. R. Mansvelder H. D. Da Lopes Silva F. H., vol. 193 (Amsterdam, Netherlands: Elsevier), 93–110. 10.1016/B978-0-444-53839-0.00007-7 21854958

[ref51] Mölle M. Born J. (2011b). Slow oscillations orchestrating fast oscillations and memory consolidation. Prog. Brain Res. 193, 93–110. doi: 10.1016/b978-0-444-53839-0.00007-7, PMID: 21854958

[ref52] Mölle M. Marshall L. Gais S. Born J. (2002). Grouping of spindle activity during slow oscillations in human non-rapid eye movement sleep. J. Neurosci. 22, 10941–10947. doi: 10.1523/jneurosci.22-24-10941.2002, PMID: 12486189 PMC6758415

[ref53] Motamedi G. K. Meador K. J. (2004). Antiepileptic drugs and memory. Epilepsy Behav. 5, 435–439. doi: 10.1016/j.yebeh.2004.03.006, PMID: 15256178

[ref54] Nayak C. S. Anilkumar A. C. (2025). EEG Normal waveforms. Treasure Island, FL: StatPearls Publishing. 30969627

[ref55] Ngo H.-V. V. Martinetz T. Born J. Mölle M. (2013). Auditory closed-loop stimulation of the sleep slow oscillation enhances memory. Neuron 78, 545–553. doi: 10.1016/j.neuron.2013.03.006, PMID: 23583623

[ref56] Norman Y. Yeagle E. M. Khuvis S. Harel M. Mehta A. D. Malach R. (2019). Hippocampal sharp-wave ripples linked to visual episodic recollection in humans. Science 365:1030. doi: 10.1126/science.aax1030, PMID: 31416934

[ref57] Plihal W. Born J. (1997). Effects of early and late nocturnal sleep on declarative and procedural memory. J. Cogn. Neurosci. 9, 534–547. doi: 10.1162/jocn.1997.9.4.534, PMID: 23968216

[ref58] Pohlack S. T. Meyer P. Cacciaglia R. Liebscher C. Ridder S. Flor H. (2014). Bigger is better! Hippocampal volume and declarative memory performance in healthy young men. Brain Struct. Funct. 219, 255–267. doi: 10.1007/s00429-012-0497-z, PMID: 23269366 PMC3889822

[ref59] Poon J. J. Y. Chapman J. L. Wong K. K. H. Mullins A. E. Cho G. Kim J. W.. (2019). Intra-individual stability of NREM sleep quantitative EEG measures in obstructive sleep apnea. J. Sleep Res. 28:e12838. doi: 10.1111/jsr.12838, PMID: 30821056

[ref60] Prehn-Kristensen A. Munz M. Göder R. Wilhelm I. Korr K. Vahl W.. (2014). Transcranial oscillatory direct current stimulation during sleep improves declarative memory consolidation in children with attention-deficit/hyperactivity disorder to a level comparable to healthy controls. Brain Stimul. 7, 793–799. doi: 10.1016/j.brs.2014.07.036, PMID: 25153776

[ref61] Rasch B. Born J. (2013). About sleep's role in memory. Physiol. Rev. 93, 681–766. doi: 10.1152/physrev.00032.2012, PMID: 23589831 PMC3768102

[ref62] Riemann D. Krone L. B. Wulff K. Nissen C. (2020). Sleep, insomnia, and depression. Neuropsychopharmacology 45, 74–89. doi: 10.1038/s41386-019-0411-y, PMID: 31071719 PMC6879516

[ref63] Robertson E. M. Pascual-Leone A. Press D. Z. (2004). Awareness modifies the skill-learning benefits of sleep. Curr. Biol. 14, 208–212. doi: 10.1016/j.cub.2004.01.027, PMID: 14761652

[ref64] Roebber J. K. Lewis P. A. Crunelli V. Navarrete M. Hamandi K. (2022). Effects of anti-seizure medication on sleep spindles and slow waves in drug-resistant epilepsy. Brain Sci. 12:288. doi: 10.3390/brainsci12101288, PMID: 36291222 PMC9599317

[ref65] Salih F. Sharott A. Khatami R. Trottenberg T. Schneider G. Kupsch A.. (2009). Functional connectivity between motor cortex and globus pallidus in human non-REM sleep. J. Physiol. 587, 1071–1086. doi: 10.1113/jphysiol.2008.164327, PMID: 19139047 PMC2673776

[ref66] Sánchez-Corzo A. Baum D. M. Irani M. Hinrichs S. Reisenegger R. Whitaker G. A.. (2024). Odor cueing of declarative memories during sleep enhances coordinated spindles and slow oscillations. NeuroImage 287:120521. doi: 10.1016/j.neuroimage.2024.120521, PMID: 38244877

[ref67] Sarkis R. A. Alam J. Pavlova M. K. Dworetzky B. A. Pennell P. B. Stickgold R.. (2016). Sleep-dependent memory consolidation in the epilepsy monitoring unit: a pilot study. Clin. Neurophysiol. 127, 2785–2790. doi: 10.1016/j.clinph.2016.05.275, PMID: 27417054 PMC5841590

[ref68] Scott H. Naik G. Lechat B. Manners J. Fitton J. Nguyen D. P.. (2024). Are we getting enough sleep? Frequent irregular sleep found in an analysis of over 11 million nights of objective in-home sleep data. Sleep Health 10, 91–97. doi: 10.1016/j.sleh.2023.10.016, PMID: 38071172

[ref69] Siapas A. G. Wilson M. A. (1998). Coordinated interactions between hippocampal ripples and cortical spindles during slow-wave sleep. Neuron 21, 1123–1128. doi: 10.1016/S0896-6273(00)80629-7, PMID: 9856467

[ref70] Snipes S. Krugliakova E. Meier E. Huber R. (2022). The theta paradox: 4-8 Hz EEG oscillations reflect both sleep pressure and cognitive control. J. Neurosci. 42, 8569–8586. doi: 10.1523/jneurosci.1063-22.2022, PMID: 36202618 PMC9665934

[ref71] Solano A. Riquelme L. A. Perez-Chada D. Della-Maggiore V. (2022). Motor learning promotes the coupling between fast spindles and slow oscillations locally over the contralateral motor network. Cereb. Cortex 32, 2493–2507. doi: 10.1093/cercor/bhab360, PMID: 34649283

[ref72] Sopp M. R. Michael T. Mecklinger A. (2018). Effects of early morning nap sleep on associative memory for neutral and emotional stimuli. Brain Res. 1698, 29–42. doi: 10.1016/j.brainres.2018.06.020, PMID: 29928870

[ref73] Spriggs W. H. (2010). Essentials of polysomnography a training guide and reference for sleep technicians. 1st Edn. Burlington, MA: Jones and Bartlett.

[ref74] Staresina B. P. Niediek J. Borger V. Surges R. Mormann F. (2023). How coupled slow oscillations, spindles and ripples coordinate neuronal processing and communication during human sleep. Nat. Neurosci. 26, 1429–1437. doi: 10.1038/s41593-023-01381-w, PMID: 37429914 PMC10400429

[ref75] Troester M. M. Quan S. F. Berry R. B. American Academy of Sleep (2023). The AASM manual for the scoring of sleep and associated events: Rules, terminology and technical specifications. 3rd Edn. Darien, IL: American Academy of Sleep Medicine.

[ref76] Tulving E. (1972). “Episodic and semantic memory” in Organization of memory. Eds. E. Tulving and W. Donaldson (Cambridge, MA: Academic Press).

[ref77] van Schalkwijk F. J. Ricci M. Nikpour A. Miller L. A. (2018). The impact of sleep characteristics and epilepsy variables on memory performance in patients with focal seizures. Epilepsy Behav. 87, 152–158. doi: 10.1016/j.yebeh.2018.06.034, PMID: 30097340

[ref78] Wagner U. Gais S. Haider H. Verleger R. Born J. (2004). Sleep inspires insight. Nature 427, 352–355. doi: 10.1038/nature02223, PMID: 14737168

[ref79] Watson N. F. Badr M. S. Belenky G. Bliwise D. L. Buxton O. M. Buysse D.. (2015). Recommended amount of sleep for a healthy adult: a joint consensus statement of the American Academy of sleep medicine and Sleep Research Society. Sleep 38, 843–844. doi: 10.5665/sleep.4716 26039963 PMC4434546

[ref80] Wilhelm I. Rose M. Imhof K. I. Rasch B. Büchel C. Born J. (2013). The sleeping child outplays the adult's capacity to convert implicit into explicit knowledge. Nat. Neurosci. 16, 391–393. doi: 10.1038/nn.3343, PMID: 23434910

[ref81] World Health Organisation (2024). Ageing and Health. Available online at: https://www.who.int/news-room/fact-sheets/detail/ageing-and-health (Accessed April 04, 2025).

[ref82] Wunderlin M. Züst M. A. Hertenstein E. Fehér K. D. Schneider C. L. Klöppel S.. (2021). Modulating overnight memory consolidation by acoustic stimulation during slow-wave sleep: a systematic review and meta-analysis. Sleep 44:296. doi: 10.1093/sleep/zsaa296, PMID: 33406249

[ref83] Xia T. Yao Z. Guo X. Liu J. Chen D. Liu Q.. (2023). Updating memories of unwanted emotions during human sleep. Curr. Biol. 33, 309–320.e5. doi: 10.1016/j.cub.2022.12.004, PMID: 36584677 PMC9979073

[ref84] Yordanova J. Kolev V. Bruns E. Kirov R. Verleger R. (2017). Sleep spindles in the right hemisphere support awareness of regularities and reflect pre-sleep activations. Sleep 40:zsx151. doi: 10.1093/sleep/zsx151, PMID: 28958008 PMC5806558

[ref85] Yordanova J. Kolev V. Verleger R. Bataghva Z. Born J. Wagner U. (2008). Shifting from implicit to explicit knowledge: different roles of early-and late-night sleep. Learn. Mem. 15, 508–515. doi: 10.1101/lm.897908, PMID: 18626095 PMC2505318

[ref86] Yordanova J. Kolev V. Wagner U. Born J. Verleger R. (2012). Increased alpha (8–12 Hz) activity during slow wave sleep as a marker for the transition from implicit knowledge to explicit insight. J. Cogn. Neurosci. 24, 119–132. doi: 10.1162/jocn_a_00097, PMID: 21812555

[ref87] Zeller C. J. Wunderlin M. Wicki K. Teunissen C. E. Nissen C. Züst M. A.. (2024). Multi-night acoustic stimulation is associated with better sleep, amyloid dynamics, and memory in older adults with cognitive impairment. Geroscience 46, 6157–6172. doi: 10.1007/s11357-024-01195-z, PMID: 38744792 PMC11493878

[ref88] Zhou Z. Norimoto H. (2023). Sleep sharp wave ripple and its functions in memory and synaptic plasticity. Neurosci. Res. 189, 20–28. doi: 10.1016/j.neures.2023.01.011, PMID: 37045494

[ref89] Zohuri B. McDaniel P. (2022). Chapter 5 - sleep driving improvement of declarative memory. Zohuri B. McDaniel P. (Zohuri B. McDaniel P. Eds.), Transcranial magnetic and electrical brain stimulation for neurological disorders (251–266). Cambridge, MA: Academic Press.

